# On inductive biases for the robust and interpretable prediction of drug concentrations using deep compartment models

**DOI:** 10.1007/s10928-024-09906-x

**Published:** 2024-03-26

**Authors:** Alexander Janssen, Frank C. Bennis, Marjon H. Cnossen, Ron A. A. Mathôt, for the OPTI-CLOT Study Group and SYMPHONY Consortium

**Affiliations:** 1 Department of Clinical Pharmacology, Hospital Pharmacy, Amsterdam UMC, University of Amsterdam, Amsterdam, The Netherlands; 2 Follow Me & Emma Neuroscience Group, Emma Children’s Hospital, Amsterdam UMC, University of Amsterdam, Meibergdreef 9, Amsterdam, The Netherlands; 3 Amsterdam Reproduction and Development, Amsterdam, The Netherlands; 4 Department of Pediatric Hematology, Erasmus MC Sophia Children’s Hospital, Erasmus University Medical Center Rotterdam, Rotterdam, The Netherlands

**Keywords:** Machine learning, Pharmacokinetics, Neural networks, Interpretability, Neural-ODE

## Abstract

Conventional pharmacokinetic (PK) models contain several useful inductive biases guiding model convergence to more realistic predictions of drug concentrations. Implementing similar biases in standard neural networks can be challenging, but might be fundamental for model robustness and predictive performance. In this study, we build on the deep compartment model (DCM) architecture by introducing constraints that guide the model to explore more physiologically realistic solutions. Using a simulation study, we show that constraints improve robustness in sparse data settings. Additionally, predicted concentration–time curves took on more realistic shapes compared to unconstrained models. Next, we propose the use of multi-branch networks, where each covariate can be connected to specific PK parameters, to reduce the propensity of models to learn spurious effects. Another benefit of this architecture is that covariate effects are isolated, enabling model interpretability through the visualization of learned functions. We show that all models were sensitive to learning false effects when trained in the presence of unimportant covariates, indicating the importance of selecting an appropriate set of covariates to link to the PK parameters. Finally, we compared the predictive performance of the constrained models to previous relevant population PK models on a real-world data set of 69 haemophilia A patients. Here, constrained models obtained higher accuracy compared to the standard DCM, with the multi-branch network outperforming previous PK models. We conclude that physiological-based constraints can improve model robustness. We describe an interpretable architecture which aids model trust, which will be key for the adoption of machine learning-based models in clinical practice.

**Supplementary Information:**

The online version contains supplementary material available at 10.1007/s10928-024-09906-x.

## Introduction

Selection of appropriate drug dosage is an important aspect underlying the efficacy of treatment and the prevention of drug-induced toxicity. However, selecting optimal doses on an individual basis can be challenging, which has historically led most to follow weight-based dosing regimens. It has frequently been reported that these conventional regimens can result in considerable inter-individual variability of achieved drug concentrations [1–3]. For example, in a study of haemophilia A patients receiving factor VIII (FVIII) concentrate, weight-based dosing (50 IU/kg) was observed to result in as high as a tenfold variation in peak FVIII levels [1]. Such large discrepancies could be especially concerning during surgical procedures, where maintaining appropriate FVIII levels is thought to be important for reducing the risk of (severe) bleeding [4]. Previous studies have shown that personalization of treatment based on the individual pharmacokinetic (PK) profile of the patient resulted in improved achievement of target FVIII levels during the perioperative setting compared to weight-based dosing [4].

Population PK involves the study of inter-individual differences in drug absorption, distribution, metabolism, and elimination [5]. PK models leverage mathematical representations of these processes to predict in vivo drug concentrations. PK models estimate a set of latent variables (PK parameters; which for example represent drug clearance or volume of distribution) based on covariate data using hand-picked closed-form expressions describing covariate effects. These estimates are then fed into a system of differential equations—so-called compartment models—which encode prior knowledge of drug distribution [6]. Considerable time and expertise is required for the development of population PK models, partly due to the manual selection of covariates and fine-tuning of the functions describing their effect. Another downside of the classical approach is that more complex and unconventional functions are rarely considered in favour of linear or power functions. This might hurt the predictive performance of such methods. Finally, development of population PK models rarely involves internal or external validation procedures, and the use of simple covariate effects and significance testing of model components might mask risks of overfitting and poor generalizability.

Recently there has been increased interest in the use of machine learning (ML) based approaches for performing PK analysis [7]. Several methods have been suggested to screen covariates based on feature importance [30, 31] or to inform function selection [32, 33]. Population PK models can also directly leverage ML methods which has the potential to improve model accuracy while reducing time spend on model development by for example directly learning drug kinetics [8] or covariate implementation [9] from data. However, the design of a reliable approach in the context of pharmacometrics is non-trivial: drug concentration data is often sparsely and irregularly sampled, while treatment interventions (e.g. drug administration) can be notably different between individuals. Additionally, we wish to use these models to evaluate counterfactual scenarios (evaluating different treatment strategies) meaning that these models should reliably extrapolate to unseen data. It might therefore be necessary to include prior knowledge into model structure to allow for more data-efficient learning. Most ML methods are also prone to overfitting, so it might be difficult for physicians to place their trust in these methods without some form of interpretability or prediction uncertainty [25, 26]. The European Commission’s proposed Regulation on Artificial Intelligence also explicitly places such requirements on ML models before they can be used for healthcare applications (AI Act recital 47, https://www.euaiact.com/recital/47, accessed 19 December 2023). A potential positive consequence of these requirements might be that ML-based algorithms will be more extensively validated compared to classical methods.

### Inductive biases

In population PK models, three sources of inductive biases help to improve model convergence: the structure of the compartment model, the equations chosen to represent covariate effects, and the use of informed initial estimates of model parameters. In contrast, naive neural networks encode weak inductive biases for dealing with tabular or time-series data. It can be shown that naive neural networks incorrectly handle important variables such as dose leading to incorrect extrapolation [7]. This problem is inherent to the inclusion of dose as a model input and is likely equally problematic in other standard ML methods (e.g. random forests and gradient boosting) [8]. Importantly, Lu et al. have even shown how neural network architectures specialized for time series predictions such as recurrent neural networks (RNNs) and long short-term memory models (LSTMs) fail to reliably extrapolate to unseen dosing schedules [8]. These limitations cannot be overcome without a causal use of variables such as dose, which potentially necessitates the use of ordinary differential equation (ODE) based methods [29]. Neural-ODE-based approaches, where treatment directly affects the latent state of the model at discrete time points, indeed do correctly respond to new and complex dosing regimens. These models are fully data-driven, greatly simplifying model development. Multiple Neural-ODE-based approaches have been suggested which can be used to learn unknown parts of the dynamical system [10, 27], or augment expert models by learning latent effects [11]. The deep compartment model (DCM) approach by [9] uses neural networks to predict the PK parameters for a compartment model, implementing doses as time-based events directly affecting drug concentrations in specific compartments. This approach has the benefit of predicting the same variables used in PK models allowing for the comparison of results. Additionally, prior knowledge on drug kinetics can be included through the compartment model, potentially improving data efficiency.

**Fig. 1:**
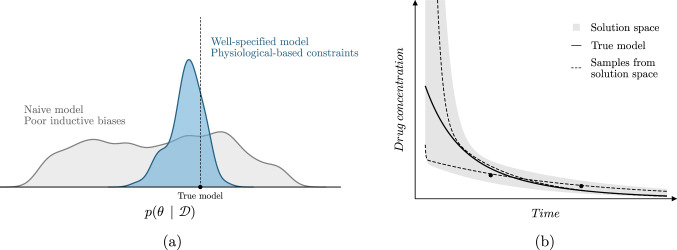
Schematic representation of the solution space of naive and well-specified models. In **a** we show the solution space of naive and well-specified models. In **b** the solution space of a model with poor inductive biases is shown. Samples from the solution space (dashed lines) can be physiologically unrealistic when data is sparse, and can differ greatly with the true solution (solid line)

Explicitly learning the dynamical system underlying observations likely serves as a useful inductive bias to improve the reliability of predictions. However, as drug concentration measurements are often sparse, the solution space given the data of potential models for these ODE-based methods can still be considerably large (see Fig. 1). As a result, unconstrained models might place similar likelihood on many different model parametrizations (Fig. 1a). Alternatively, well-specified models with physiological-based constraints result in more concentrated posterior distributions. If these biases are well-adjusted and informative, the resulting posterior might be more similar to the true model. In Fig. 1b, we depict an example of models within the solution space of unconstrained models. Some of the models might learn potentially physiologically implausible or unlikely concentration–time curves (dashed lines).

In this work, we introduce simple inductive biases within the deep compartment model framework by placing domain-specific constraints on model architecture to improve robustness. We define models as not robust if they have a high propensity of learning spurious effects. We investigated effects on model accuracy and stability of providing bounds for the value of the PK parameters, estimating global values for difficult to identify parameters, and connecting covariates to specific PK parameters. Expanding on the latter constraint, fully-connected neural networks encode an implicit assumption that part of the signal potentially originates from complex interactions between the covariates. Model generalizability and robustness can potentially be improved by only linking covariates with causal effects to specific PK parameters in sub-models. An additional benefit of this approach is that the learned function from each sub-model can be visualized, enabling model interpretation.

## Methods

### Problem definition

Our focus is on haemophilia A, a blood clotting disorder where a deficiency of FVIII results in elevated (spontaneous) bleeding risk. Haemophilia A patients are treated by intravenous injection of FVIII at regular intervals. The PK of FVIII is often described using a two-compartmental structure, where the first compartment represents the distribution of FVIII into the blood and the second is often thought to represent the initial rapid clearance of FVIII or its binding to intra or extra-vascular space [12–14]. The two-compartmental model can be represented by the following system of partial differential equations:

1 $$\begin{array}{*{20}c} {\frac{{dA_{1} }}{dt}} =& {\frac{I}{{V_{1} }} + A_{2} \cdot k_{21} - A_{1} \left( {k_{10} + k_{12} } \right)} \\ {\frac{{dA_{2} }}{dt}} =& {A_{1} \cdot k_{12} - A_{2} \cdot k_{21} } \\ \end{array}$$

Here, the rate constants $k$ describe the flow between the compartments specified in its subscript, ${A}_{1}$ represents the concentration in the 1st compartment (and so on), and $I$ represents the rate of drug entering the first compartment after drug administration. The rate constants $k$ are functions of the PK parameters: ${k}_{10}=\frac{CL}{{V}_{1}}$, ${k}_{12}=\frac{Q}{{V}_{1}}$, and ${k}_{21}=\frac{Q}{{V}_{2}}$ with $z=\{CL,Q,{V}_{1},{V}_{2}\}$ referring to clearance, inter-compartmental clearance, central distribution volume, and peripheral distribution volume, respectively.

Consider a population of $n$ individuals with $\mathcal{D}={\left({x}^{\left(i\right)},{t}^{\left(i\right)},{y}^{\left(i\right)}\right)}_{i\in \left[1..n\right]}$, each with irregular drug concentration measurements $y^{\left( i \right)} \in {\mathbb{R}}_{+}^{K}$ sampled over time horizon ${t}^{\left(i\right)}\in \left[0,{T}_{i}\right]$ with ${T}_{i}$ indicating the follow-up time for individual $i$. Drug concentration predictions are produced based on the compartment model and a matrix of interventions ${I}^{\left(i\right)}$ containing information on time of dose, dosage, and infusion rates that affect the integrator at the specified time points:

2 $$\hat{y}^{\left( i \right)}(t) = A\left( {t;z^{\left( i \right)} ,I^{\left( i \right)} } \right)$$

In non-linear mixed effects models, individual estimates of each of the PK parameters $z^{\left( i \right)} \in {\mathbb{R}}_{+}^{M}$ are obtained based on covariates ${x}^{\left(i\right)}\in {\mathbb{R}}^{D}$ and subject-specific random effects ${\eta }^{\left(i\right)}\sim N\left(0,\Omega \right)$, where $\Omega$ is a $M\times M$ covariance matrix when random effects are included on all PK parameters. The following implementation is frequently observed within the pharmacometrics literature:

3 $$z_{m}^{\left( i \right)} = \theta_{m} \cdot \exp \left( {\eta_{m}^{\left( i \right)} } \right) \cdot \mathop \prod \limits_{s}^{{S_{m} }} f_{s} \left( {x_{s} ;\theta_{s} } \right)$$

Here, $\theta$ represent model fixed effect parameters and ${S}_{m}\subset \left[1..D\right]$ indicates the subset of covariates used to predict ${z}_{m}$. After specifying a model for the residual error $\epsilon \sim N\left(0,\Sigma \right)$ on ${y}^{\left(i\right)}$ (e.g. additive, proportional, or a combination of both), model parameters $\Theta =\{\theta ,\Omega ,\Sigma \}$ can be optimized by maximizing:

4 $$\hat{\Theta } = \arg \max_{\Theta } {\mathcal{L}}\left( \Theta \right) = \mathop \prod \limits_{i = 1}^{N} p\left( {y^{\left( i \right)} |\Theta ,\eta^{\left( i \right)} } \right)p\left( {\eta^{\left( i \right)} } \right)$$

As previously mentioned, development of non-linear mixed effects models requires considerable time and expertise, partly due to the manual selection of covariates and the functions $f$ to represent their effect on $z$. In DCMs, the fixed effect model is learned by a neural network $\phi$ with parameters $w$, and the covariates are used to predict typical PK parameters ${\zeta }^{\left(i\right)}$:

5 $$\zeta^{\left( i \right)} = \phi \left( {x^{\left( i \right)} ;w} \right)$$

And the model minimizes the squared error:

6 $$\hat{w} = \arg \min_{w} {\mathcal{L}}\left( w \right) = \mathop \sum \limits_{i = 1}^{N} \mathop \sum \limits_{k = 1}^{{K_{i} }} \left( {y_{k}^{\left( i \right)} - A\left( {t_{k}^{\left( i \right)} ;\zeta^{\left( i \right)} ,I^{\left( i \right)} } \right)} \right)^{2}$$

These models are relatively unconstrained in their prediction of ${\zeta }^{\left(i\right)}$, as long as it results in low error with respect to the observations. It can thus be the case that the model is not penalized for making extreme predictions outside of the observed data.Model constraints.

**Fig. 2:**
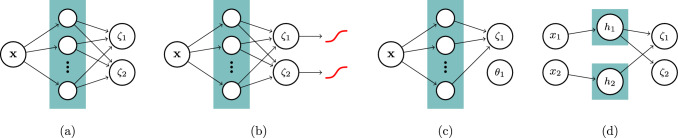
Graphical models representing the model structure of the proposed architectures. Naive (**a**), boundary constraint (**b**), global parameter (**c**), and multi-branch network (**d**) architectures are depicted. Nodes represents neurons, with the coloured box representing the hidden layer of the neural network

We propose three simple approaches for constraining the solution space of DCMs (Fig. 2). First, boundary conditions were imposed on the PK parameters by using a transformed sigmoidal function following the output layer of the neural network (referenced as *boundary* constraint; Fig. 2b). The boundaries can be set empirically based on prior knowledge. For example, bounds for the volume of distribution of drugs tightly bound to plasma proteins can be based on the expectation that the plasma volume of a typical male is roughly around 46—52 mL/kg [15]. Lower bounds of [0, 0.3, 0.05, 0] and upper bounds of [0.5, 7, 0.5, 2] for respectively $CL$ (L/h), ${V}_{1}$ (L), $Q$ (L/h), and ${V}_{2}$ (L) were used.

Next, global parameters $\theta$ for a subset of the PK parameters were estimated in parallel to $w$ (referenced as *global parameters* constraint; Fig. 2c). We chose to estimate $\theta =\{Q,{V}_{2}\}$ since these parameters affect the early distribution of FVIII, and drug concentration measurements at early time points are usually too sparse to identify covariate effects on these parameters.

Finally, we describe a neural network architecture where each covariate (or specific combinations thereof) are connected to specific PK parameters via independent sub-models, whose predictions are combined using a product (referenced as the *multi-branch network*; Fig. 2d). This architecture is similar to a generalized additive model, using product accumulation rather than the sum of covariate effects. The use of a product matches the standard implementation of covariates in population PK models (Eq. 3), and facilitates the interpretation of the clinical relevance of each covariate. For example, covariates resulting in a maximal net change 20% of the corresponding PK parameter are often deemed clinically insignificant in the pharmacometrics literature [16]. An additional benefit of the approach is that the output of each sub-model can be visualized, allowing for the interpretation of the learned covariate effects. A schematic overview of the multi-branch network is provided in Supplementary Fig. 1.

More details about the specific implementation of the model constraints can be found in Supplementary Data 1A.

### Synthetic experiments

#### Data generation

We simulated a data set of haemophilia A patients based on population data from the American National Health and Nutrition Examination Survey (NHANES) [17]. The weight, height, and age of 756 male individuals without missing data were collected from the data set and used as covariates. FVIII levels were simulated based on an existing population PK model of an extended half-life FVIII concentrate [18]. This model was chosen as it used an estimate of the fat-free mass (FFM) from [19] to predict FVIII clearance ($CL$) and central volume of distribution (${V}_{1}$):

7 $$FFM = \left( {0.88 + \frac{1 - 0.88}{{1 + \frac{Age}{{13.4}}^{ - 12.7} }}} \right) \cdot \left( {\frac{9270 \cdot Weight}{{6680 + \left( {216 \cdot BMI} \right)}}} \right)$$

This allowed for the comparison of model accuracy when training models using FFM directly as well as using its components weight, height (as part of BMI), and age. This could give an indication of the accuracy at which non-linear interactions of covariates could be learned.

The previous PK model was based on a two-compartment model, with inter-individual variability on the $CL$ and ${V}_{1}$ parameters. Using the structural equations reported by [18], typical estimates of the PK parameters were produced. Next, samples of the random effects were drawn to produce individual estimates of the PK parameters. Each individual received a dose of 50 IU/kg, rounded to the nearest 250 IU. To allow for stochasticity of measurement times, samples were taken from a multivariate normal distribution ${t}^{\left(i\right)}\sim N\left(\left[\mathrm{4,24,48}\right],\sigma =\left[\mathrm{2,5},5\right]\right)$. Sampling times were truncated at $t=0.25$ (i.e. 15 min after dose) to prevent samples at negative time points or too close to the time of dose administration. Finally, the ODE was solved based on the individual PK parameters to simulate FVIII levels for each individual and additive error ($\sigma =5.0$ IU/dL) was added to create the training data.

### Evaluation of model constraints

Prediction accuracy of the proposed constraints was compared to a naive neural network as well as the initialization approach suggested in [9]. In all experiments, covariates were scaled between 0 and 1 using min–max scaling. Models were trained using patient weight, height and age, or FFM and age. A two compartment model was used. Each neural network was trained using a single hidden layer of either 8, 32, or 128 neurons followed by the swish activation function [20]. A softplus activation function was used in the output layer of the naive neural network as well as in the model estimating global parameters for $Q$ and ${V}_{2}$ to constrain latent variables to ${\mathbb{R}}^{+}$. All models were trained for 500 epochs using the ADAM optimizer with a learning rate of 1e-2 [21]. We found that these settings were sufficient for each model to converge before the end of optimization. First, prediction accuracy and robustness of the naive, initialization, boundary, and global parameter models were compared. The multi-branch network was not tested in this context due to similarities to the global parameter model. Each model was fit to a random subset of the simulated data of size 20, 60, or 120 to represent data sets of small, medium, and large size, respectively. A Monte Carlo cross validation of 20 different train and test sets was performed in order to estimate the stability of model predictions. In addition, model training was replicated five times on each train-test split. This resulted in a total number of 100 replicates of each model, which was deemed sufficient to estimate model variability given our computational budget. Model accuracy was represented by the root mean squared error (RMSE) of predicted FVIII levels compared to the true, simulated concentration–time curves on the test set. To this end, true and predicted FVIII levels were collected at five minute intervals until $t=72h$ as a means to approximate the error compared to the full concentration–time curve. Models were compared in terms of their median RMSE over the 20 data sets and five model replicates. Model robustness for each of the architectures was represented by the percentage of models with RMSE greater than 150% of the median RMSE (references as divergent models).

In order to evaluate differences between using fully-connected versus multi-branch neural networks, the data set was augmented with two continuous and one categorical covariate without correlations to the other covariates. For the continuous covariates, random samples were drawn from Uniform(0,1) distributions, while for the categorical covariate samples were randomly assigned to one of five categories with equal probability. Next, three models with global $Q$ and ${V}_{2}$ parameters were fit to FFM, age, and the noise covariates: (1) a fully-connected model, (2) a multi-branch network with all covariates independently connected to $CL$ and ${V}_{1}$, and (3) a multi-branch network with the ground truth covariate connections as used in the simulation (referenced as the *causal model*). Fully-connected models were trained using a single hidden layer of 32 neurons. The number of neurons in the hidden layer of each sub-model was set to 16 to ensure that models had roughly similar number of parameters. Accuracy was again compared using the RMSE. Results were compared with the global parameter models trained on FFM and age from the first experiment (models trained using 32 neurons). Covariate effects from the multi-branch network were visualized to facilitate model interpretation (See Supplementary Data 2 for implementation details).

All model code and synthetic data will be made available at https://github.com/Janssena/dcm-constrained.

### Real-world experiments

We compared the predictive performance of two previously published population PK models [22, 23] to the DCM with or without the proposed constraints and a Neural-ODE based model [8]. Data consisted of 69 severe haemophilia A patients who received a single dose of 25–50 IU/kg standard half-life FVIII. For each patient, three measurements were available roughly 4, 24, and 48 h after dose. Available covariates without missing data were patient weight, height, age, and blood group.

The population PK model by [14] included the effect of weight on all PK parameters, as well as the effect of age on $CL$. The model implemented allometric scaling, which is very common in PK models. We also evaluated the performance of a more recent model by [23]. Instead of using weight, this model implements the effect of FFM on clearance and volume of distribution. An effect of patient age was also included on clearance. Since it is well documented that patients with blood group O have higher FVIII $CL$ compared to non-O patients, we also fitted models including a proportional effect of having blood group O on $CL$ [24].

DCMs were fit using neural networks with a single layer containing 8, 32 or 128 neurons (halved for each sub-model for the multi-branch network). For the fully-connected network, model input was patient weight, height, age, and BGO. Models were fit without constraints, using boundary constraints (same as used during simulation experiments), and using global parameters for $Q$ and ${V}_{2}$. In the multi-branch network clearance was predicted based on patient a combination of weight and height, age, and BGO, while estimating volume of distribution based on a combination of weight and height. Global parameters were estimated for $Q$ and ${V}_{2}$ in all models.

For the Neural-ODE based model we followed the general architecture by Lu et al. [8]. Hyper-parameters were the number of neurons in the encoder, Neural-ODE, and decoder (8, or 32), the number of hidden layers (1 or 2) in the Neural-ODE, and the number of the latent variables (2 or 6). Encoder and decoder consisted of a single hidden layer. Tanh activation functions were used in the Neural-ODE to improve model stability. Model input was patient weight, height, age, and BGO and values were normalized between − 1 and 1. This marks an important difference to the model by Lu et al., where part of the drug concentration measurements were used as input to the encoder and decoder.

### Model training and evaluation

A ten-fold cross-validation was performed for both the non-linear mixed effects models and DCMs. Both PK models were implemented in the NONMEM software (ICON Development Solutions, Ellicott City, MD) and model parameters were re-estimated on each full train fold. Exponents of the effects of weight on the PK parameters were not re-estimated in the model by [14] since they follow the concept of allometric scaling. The accuracy of typical predictions were reported. The ML models were trained for 4000 epochs which was more than sufficient for model convergence, and neural network weights resulting in the lowest validation error (20% of training fold) were saved. Hyper-parameter selection was performed by comparison of the RMSE on the validation sets. Results for the models with lowest average validation error were presented. The average RMSE of predictions with respect to the test fold was reported.

## Results

### Constraints improve model robustness

In the first experiment, highest model accuracy was generally obtained when using a hidden layer size of 8 neurons (see Table 1). Results for models trained with larger hidden layer sizes can be found in Supplementary Tables 1 and 2. All models seemed to perform similarly well when sufficient data was available. When training on smaller data-sets, the median RMSE and its variance increases for all models. However, when training naive models, a relevant proportion of models (18%) presented with a highly divergent error on the small data set (mean RMSE 44.4 IU/dL).

**Table 1:** Test set accuracy and divergence rate for models with a hidden layer size of 8. Median RMSE over all replicates of model training (5 $\times$ 20 data sets) during experiment 1 is reported along with its standard deviation

Median RMSE ± one SD (%-age divergent)			
	$n=20$	$n=60$	$n=120$
Weight, height, age			
None	14.6 ± 14 (18)	13.1 ± 1.2 (0)	12.3 ± 0.34 (0)
Initialization	15.3 ± 21 (6)	12.9 ± 2.0 (2)	12.0 ± 0.45 (0)
Boundary	14.9 ± 2.5 (3)	12.6 ± 0.55 (0)	12.0 ± 0.45 (0)
Global parameters	13.9 ± 0.94 (0)	12.9 ± 0.44 (0)	12.3 ± 6.0 (1)
FFM, age			
None	14.1 ± 10 (12)	12.8 ± 0.71 (0)	12.2 ± 0.39 (0)
Initialization	14.2 ± 16 (6)	12.5 ± 1.2 (2)	11.9 ± 0.3 (0)
Boundary	13.8 ± 1.2 (0)	12.4 ± 0.33 (0)	11.9 ± 0.3 (0)
Global parameters	13.5 ± 0.75 (0)	12.6 ± 0.35 (0)	12.2 ± 0.38 (0)

*RMSE* root mean squared error, *SD* standard deviation

In contrast, model accuracy was more stable when using model constraints, with only two divergent models (0.33%) over all models with global parameters (including those with larger hidden layer sizes). Setting boundary constraints reduced the number of divergent models compared to the previously suggested approach of initialization, but was less effective compared to the global parameter model (nine divergent models overall). Looking at the naive models fit with 128 neurons, both model accuracy and robustness was negatively affected when training at lower sample sizes (Supplementary Table 1). Median RMSE for the naive model trained on 20 samples increased from 14.7 to 16.5 IU/dL when changing hidden layer size from 8 to 128 neurons. In contrast, models fit using global parameters were almost unaffected by hidden layer size in the same context (RMSE 13.9 to 14.1 IU/dL). Models trained using FFM and age resulted in slightly more accurate predictions when trained on 20 samples, with almost no differences in medium to large data sets. A more extensive investigation of the effect of the constraints on model training can be found in Supplementary Data 1B. Here, we found that divergent behaviour was specific to certain data folds, and was related to the estimate of V_2._ Adding constraints to this specific parameter was sometimes sufficient to improve models.

**Fig. 3:**
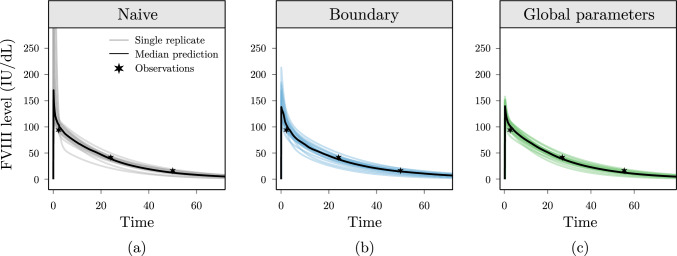
Predicted concentration–time curves from the proposed constraints are more realistic compared to naive models. Results are shown for the naive (**a**), boundary constraint (**b**), and global parameter (**c**) model. The median prediction (black line) over the 20 data set replicates (lightly coloured lines) along with the observations (stars) are shown for the same patient

Next, we inspected the predicted concentration–time curves from each model. In Fig. 3, we show the predictions for a single, representative patient for naive (a), boundary (b), and global parameter (c) models. Here, we see that all models accurately predict the three observed FVIII levels. However, the naive model seems biased to predict unrealistically high FVIII peak levels (with predictions for some patients as high as 1340 IU/dL). In contrast, the constrained models resulted in less extreme and more similar solutions.

### False covariates degrade model performance

We then compared the fully-connected and multi-branch networks on the augmented data set (see Table 2). The addition of false covariates degraded the accuracy of the fully-connected network with global parameters when using small data sets compared to the first experiment (RMSE of 18.1 vs. 13.3 IU/dL). We found that, initially, the multi-branch network using all covariates depicted high error in several replicates (RMSE > 40 IU/dL). In these replicates, poor initializations resulted in initial ${V}_{1}$ estimates close to zero, resulting in high peak predictions as seen in Fig. 3a. To solve this issue, we initialized the bias of the neuron connecting to ${V}_{1}$ in the final layer of each sub-model to 0.5, increasing initial estimates close to 1 L. The resulting model performs slightly better at low sample sizes compared to the fully-connected network (RMSE 15.6 vs. 18.1 IU/dL). This suggests that part of the decrease in accuracy of the fully-connected network might be related to the model learning spurious interactions between the covariates.

**Table 2:** Introduction of noise covariates deteriorates accuracy of fully-connected networks. Median RMSE of the test set over all replicates of model training (5 $\times$ 20 data sets) is reported along with its standard deviation

Model	Median RMSE ± one SD (%-age divergent)		
	$n=20$	$n=60$	$n=120$
Fully-connected	18.1 ± 2.9 (2)	13.6 ± 0.52 (0)	12.7 ± 0.35 (0)
Multi-branch	15.6 ± 2.9 (1)	12.8 ± 0.48 (0)	12.1 ± 0.19 (0)
Causal	13.3 ± 1.0 (0)	12.5 ± 0.34 (0)	12.1 ± 0.25 (0)

*RMSE* root mean squared error, *SD* standard deviation

**Fig. 4:**
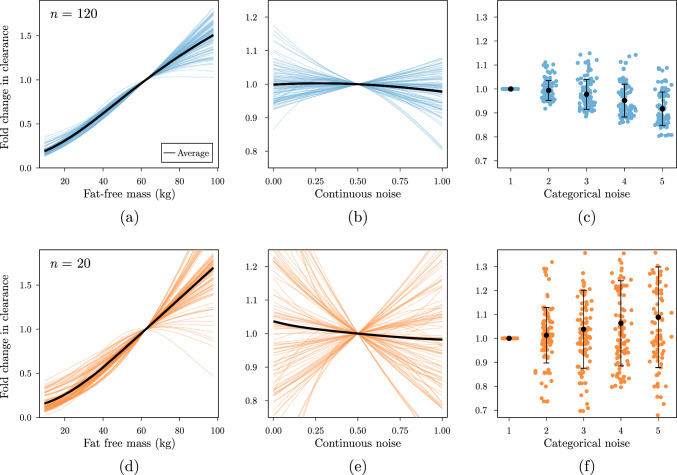
Visualization of learned functions from the multi-branch network enable model interpretability. The top panel (**a**, **b**, and **c**) depict the learned functions for the model trained on 120 samples, with the bottom panel (**d**, **e**, and **f**) showing results on $n=20$. Black curves depict the average effect over the 100 model replicates (colored lines)

By including only true effects, the causal model achieved very similar accuracy to the global parameter model from the first experiment at all data set sizes. In Fig. 4 we depict the learned covariate effects for the network containing noise covariates. As the number of training samples decreases, the variance of learned functions across replicates seemed to increase (i.e. the functions became more diverse). When trained on $n=20$, the effect of the noise covariates on clearance was quite substantial in some replicates. It is still possible to identify these covariates as unimportant overall, as their mean effect over replicates is close to 1.

### Constrained models perform better on real-world data

In Table 3, we summarize the results from the models fit to real data. Here, we see that the addition of the effect of blood group O on CL improved accuracy of the expert models. Addition of the covariate resulted in a statistically significant decrease in objective function value by more than 20 points in both models ($p<0.01$; ${\chi }^{2}$ = 6.635). The model by [23] was more accurate on our data set than the model from [14] (RMSE 13.7 vs 14.8 IU/dL). The addition of model constraints improved accuracy compared to the naive DCM, and the use of boundary constraints and global parameters resulted in models with relatively similar performance to the best performing NLME model. The multi-branch network achieved the lowest RMSE overall (13.0 IU/dL) while the Neural-ODE based model achieved the highest RMSE (19.5 IU/dL). Similar to Fig. 3, the naive model predicted higher FVIII peak levels, followed by an initial rapid re-distribution of FVIII (see Supplementary Fig. 2a). The addition of model constraints resulted in more smooth concentration time curves. Concentration time-curves produced by the Neural-ODE were unrealistic and suggestive of model overfitting (Supplementary Figs. 2e and 3). Again, learned covariate effects in the multi-branch network were visualized, enabling model interpretation (see Supplementary Fig. 4).

**Table 3:** Comparison of model accuracy on real-world data

	Mean RMSE (IU/dL) ± one SD
Expert models	
Björkman et al. [14]	15.8 ± 3.3
Björkman et al. [14] + BGO	14.8 ± 3.1
McEneny-King et al. [23]	14.7 ± 2.9
McEneny-King et al. [23] + BGO	13.7 ± 3.0
Machine learning models	
‍ Neural-ODE (32 neurons, 1 hidden layer, 2 latent variables)	19.5 ± 3.5
Fully-connected DCM (32 neurons)	14.8 ± 2.8
DCM + boundary (32 neurons)	14.1 ± 2.4
DCM + global parameters (32 neurons)	13.9 ± 1.8
Multi-branch network (16 neurons)	13.0 ± 2.1

*RMSE* root mean squared error, *SD* standard deviation, *BGO* blood group O, *DCM* deep compartment model

## Discussion

In this work, we investigated how model constraints affected the predictive performance of deep compartment models [9]. Without any constraints, models potentially learn unrealistic concentration–time curves when data is sparse. Although these models accurately predicted observed concentration measurements (i.e. had low training loss), they were not penalized for making extreme predictions at time points outside the training data. The results from the first experiment indicated that roughly one-fifth of fitted models resulted in divergent results when data was sparse (*n* = 20). This limits the clinical implementation of such algorithms. Our results indicate that the introduction of simple constraints improved model robustness as represented by the number of divergent models. As a consequence, the constraints could improve model accuracy when trained on smaller data sets and resulted in more realistic concentration–time curves. The constraints can be based on prior knowledge, making them easier to implement in practice. Finally, the proposed multi-branch network architecture is an interpretable alternative to fully-connected networks, trading ease-of-implementation for increased model trust.

In the second synthetic experiment we found that the presence of false covariates affected model accuracy. Visualizations of the learned functions in the multi-branch network indicated that models were sensitive to learning false effects irrespective of the size of the training set. However, models trained on sparse data were more likely to inflate the importance of the false covariates, resulting in higher error on new data. This is indicative of the importance of the careful selection of (causal) covariates to include in these models. One approach for covariate selection can for example be the use of cross-validation based procedures to identify covariates that can be removed based on the uncertainty/absence of their effect across replicates. Similarly, this approach can be used to perform an initial screening of the covariates for downstream model analysis. This approach can both identify covariate importance as well as their relationship to the PK parameters. Comparing learned functions from multiple replicates also allows for the identification of regions of covariate space that have higher data uncertainty. For example, < 2% of patients had a FFM in the dataset, which is reflected by higher uncertainty of the effect of FFM on CL (Fig. 4a). For patients in these regions one can decide to first collect more data before making predictions. Knowing when to trust model predictions is important, especially in the context of medical decision-making.

The results of the real data experiment support the findings of the synthetic experiments. The addition of model constraints improved model performance in terms of test set accuracy. Importantly, the shape of the resulting concentration–time curves were again more realistic compared to those from unconstrained models. By understanding how inductive biases are encoded in conventional methods used for PK analysis, we show that hybrid architectures can be a promising approach for improving model performance in settings with limited data. Fully ML-based architectures, such as the Neural-ODE, greatly simplify model development but suffer when data is sparse. In addition, diagnosing and resolving overfitting issues in these models is more complicated. We show that hybrid architectures can alleviate these issues, and can be designed in such a way that the model is inherently interpretable. This eliminates the need for (post-hoc) ML explainability methods such as SHAP, which do not necessarily offer a *true* representation of model predictions [28]. Using Neural-ODEs for learning parts of the mechanistic model can also be an interesting hybrid approach [27]. In the multi-branch network, covariates are organized into sub-models, allowing for the visualization of learned functions. Such an approach can improve model trust while also aiding with the ability to critique the model during development. Compared to classical population PK modelling, this method holds great potential for reducing the complexity of model development especially when paired with the ability to detect and manage overfitting.

There were also some limitations to this study. First of all, in the PK model used to generate the synthetic data, $Q$ and ${V}_{2}$ parameters were fixed for all individuals. This might partly explain the higher accuracy of the models estimating global parameters for these variables. However, due to data sparsity at early time-points and the addition of noise, it is not necessarily clear in what degree this affects the results. Regardless of potential biases during the synthetic experiments, the estimation of global parameters also resulted in more accurate predictions in the experiment using real world data. Next, we found that the estimation of global parameters resulted in higher accuracy compared to the use of boundaries. Inspections of model predictions showed that estimates of $Q$ and ${V}_{2}$ were often stuck in flat regions of the sigmoid during early training (Supplementary Data 1B). Resulting gradients shrink to zero, making it more difficult for the model to correct for early misspecification. This approach could thus potentially be improved by only placing boundaries on a subset of the PK parameters, by combining it with the estimation of global parameters, or by using less aggressive functions to constrain the parameters (e.g. softsign or cdf of a Cauchy(0, 2), see Supplementary Data 1B).

A limitation of the proposed multi-branch network is that poor initializations could still be prone to fitting unrealistic models. Unfortunately, placing additional constraints on this architecture is difficult as it changes model interpretation. For example, setting boundaries on the predicted values of the PK parameters in the final layer of the network breaks the interpretation of the learned functions. Another downside is that learned effects can only be visualized when the number of covariates used in each sub-model facilitates 2 or 3-dimensional visualization. Next, we evaluated only a relatively small number of hyper-parameters settings, i.e. only a single hidden layer with three options for the number of neurons. Extensive searches over appropriate hyper-parameters can be problematic, especially when data is sparse. In the real-world experiment for example, only 12 patients were used to find the optimal weights during training as well as the optimal hyper-parameters. When evaluating a large set of hyper-parameters, we risk overfitting the hyper-parameters to the validation set. A promising alternative is to perform hyper-parameter selection based on the desired complexity of the learned functions in the multi-branch network.

In the real world experiment, we compared model performance based on prediction accuracy represented by the RMSE, similar to previous studies [8, 11]. This metric might not be sufficient to fully compare the models. However, common tools for comparing population PK models, such as the Akaike and Bayesian information criterium, are not suitable for use with neural networks as they generally over-estimate model complexity when penalizing the number of parameters. Although the current results suggest improvement of models when adding constraints, more research on multiple data sets might be needed to draw conclusions.

Finally, we only evaluated the use of constraints in the context of a drug with relatively simple kinetics. How performance is affected in more complex settings was not within the scope of the current work. It is possible that the selection of appropriate constraints can be difficult in models with an extremely large number of PK parameters. Similarly, setting constraints on parameters with a more complicated interpretation can also be difficult.

Future work could investigate the implementation of more sophisticated inductive biases. It might be of interest to selectively tighten boundaries based on patient covariates. We would for example expect lower distribution volumes for children compared to adults. Other approaches could focus on placing constraints on the learned functions in the multi-branch network, for example by encouraging monotonicity at unseen values of the covariates. Maximum a posteriori estimation of the neural network weights can also be performed using prior distributions that favour less extreme functions. Alternatively, Gaussian Processes are an interesting alternative to neural networks, as they provide a more practical approach for placing priors over the functional form of the relationships. Additionally, Gaussian Processes allow for a practical method for estimating uncertainty over learned functions. Finally, a method for performing covariate selection using the multi-branch network would be of interest to aid model development.

## Conclusion

This work has focused on improving the robustness of the deep compartment model framework. The suggested model constraints can be used to improve the performance of this model class when data is sparse, which is frequently the case in the pharmacometric literature. The proposed hybrid model has many of the benefits of current ML methods used in the pharmacometrics literature, and addresses some of their main limitations. The suggested improvements further demonstrate the method as a viable alternative to classical population PK modelling.

### Supplementary Information

Below is the link to the electronic supplementary material.

Supplementary file1 (DOCX 1822 kb)

Supplementary file2 (DOCX 6 kb)

Supplementary file3 (DOCX 380 kb)

## Data Availability

All model code and simulated data will be made available at https://github.com/Janssena/dcm-constrained.

## References

[1] Björkman S, Oh M, Spotts G (2012). Population pharmacokinetics of recombinant factor VIII: the relationships of pharmacokinetics to age and body weight. Blood J Am Soc Hematol.

[2] Lankheet NA, Knapen LM, Schellens JH (2014). Plasma concentrations of tyrosine kinase inhibitors imatinib, erlotinib, and sunitinib in routine clinical outpatient cancer care. Ther Drug Monit.

[3] Roberts JA, Paul SK, Akova M (2014). DALI: defining antibiotic levels in intensive care unit patients: are current β-lactam antibiotic doses sufficient for critically ill patients?. Clin Infect Dis.

[4] van Moort I, Preijers T, Bukkems LH (2021). Perioperative pharmacokinetic-guided factor VIII concentrate dosing in haemophilia (OPTI-CLOT trial): an open-label, multicentre, randomised, controlled trial. Lancet Haematol.

[5] Sheiner LB, Ludden T (1992). Population pharmacokinetics/dynamics. Annu Rev Pharmacol Toxicol.

[6] Holz M, Fahr A (2001). Compartment modeling. Adv Drug Deliv Rev.

[7] Janssen A, Bennis FC, Mathôt RA (2022). Adoption of machine learning in pharmacometrics: an overview of recent implementations and their considerations. Pharmaceutics.

[8] Lu J, Deng K, Zhang X (2021). Neural-ODE for pharmacokinetics modeling and its advantage to alternative machine learning models in predicting new dosing regimens. Iscience.

[9] Janssen A, Leebeek FW, Cnossen MH (2022). Deep compartment models: a deep learning approach for the reliable prediction of time-series data in pharmacokinetic modeling. Pharmacomet Syst Pharmacol.

[10] Bräm DS, Nahum U, Schropp J (2023). Neural ODEs in pharmacokinetics: concepts and applications. J Pharmacokinet Pharmacodyn.

[11] Qian Z, Zame W, Fleuren L (2021). Integrating expert ODEs into neural ODEs: pharmacology and disease progression. Adv Neural Inf Process Syst.

[12] Over J, Sixma J, Bruine M (1978). Survival of 125iodine-labeled factor VIII in normals and patients with classic hemophilia. Observations on the heterogeneity of human factor VIII. J Clin Investig.

[13] Noe DA (1996). A mathematical model of coagulation factor VIII kinetics. Pathophysiol Haemost Thromb.

[14] Björkman S, Carlsson M, Berntorp E, Stenberg P (1992). Pharmacokinetics of factor VIII in humans: obtaining clinically relevant data from comparative studies. Clin Pharmacokinet.

[15] Yiengst MJ, Shock NW (1962). Blood and plasma volume in adult males. J Appl Physiol.

[16] Xu XS, Yuan M, Zhu H (2018). Full covariate modelling approach in population pharmacokinetics: understanding the underlying hypothesis tests and implications of multiplicity. Br J Clin Pharmacol.

[17] Centers for Disease Control and Prevention (CDC). National Center for Health Statistics (NCHS). National Health and Nutrition Examination Survey Data. Hyattsville, MD: U.S. Department of Health and Human Services, Centers for Disease Control and Prevention, 2009. https://wwwn.cdc.gov/nchs/nhanes/continuousnhanes/default.aspx?BeginYear=2009

[18] Chelle P, Yeung CH, Croteau SE (2020). Development and validation of a population-pharmacokinetic model for rurioctacog alfa pegol (adynovate): a report on behalf of the WAPPS-hemo investigators ad hoc subgroup. Clin Pharmacokinet.

[19] Al-Sallami HS, Goulding A, Grant A (2015). Prediction of fat-free mass in children. Clin Pharmacokinet.

[20] Ramachandran P, Zoph B, Le QV (2017) Searching for activation functions. arXiv:171005941

[21] Kingma DP, Ba J (2014) Adam: a method for stochastic optimization. arXiv:14126980.

[22] Björkman S, Folkesson A, Jönsson S (2009). Pharmacokinetics and dose requirements of factor VIII over the age range 3–74 years: a population analysis based on 50 patients with long-term prophylactic treatment for haemophilia a. Eur J Clin Pharmacol.

[23] McEneny-King A, Chelle P, Foster G (2019). Development and evaluation of a generic population pharmacokinetic model for standard half-life factor VIII for use in dose individualization. J Pharmacokinet Pharmacodyn.

[24] O’donnell J, Laffan M (2001). The relationship between ABO histo-blood group, factor VIII and von willebrand factor. Transfus Med.

[25] He Z, Zhang R, Diallo G, Huang Z, Glicksberg BS (2023). Editorial: explainable artificial intelligence for critical healthcare applications. Front Artif Intell.

[26] Amann J, Blasimme A, Vayena E, Frey D, Madai VI; Precise4Q Consortium (2020) Explainability for artificial intelligence in healthcare: a multidisciplinary perspective. BMC Med Inform Decis Mak 20(1):310 10.1186/s12911-020-01332-6 PMC7706019 33256715

[27] Bräm DS, Nahum U, Schropp J, Pfister M, Koch G (2023). Low-dimensional neural ODEs and their application in pharmacokinetics. J Pharmacokinet Pharmacodyn.

[28] Kumar I (2021). Shapley residuals: quantifying the limits of the Shapley value for explanations. Adv Neural Inf Process Syst.

[29] Rackauckas C, Ma Y, Martensen J, Warner C, Zubov K, Supekar R et al (2020) Universal differential equations for scientific machine learning. arXiv:2001.04385

[30] Keutzer L, You H, Farnoud A, Nyberg J, Wicha SG, Maher-Edwards G, et al (2022). Machine learning and pharmacometrics for prediction of pharmacokinetic data: differences, similarities and challenges illustrated with rifampicin. Pharmaceutics.

[31] Sibieude E, Khandelwal A, Hesthaven JS, Girard P, Terranova N (2021). Fast screening of covariates in population models empowered by machine learning. J Pharmacokinet Pharmacodyn.

[32] Wahlquist Y, Sundell J, Soltesz K (2023). Learning pharmacometric covariate model structures with symbolic regression networks. J Pharmacokinet Pharmacodyn.

[33] Janssen A, Hoogendoorn M, Cnossen MH, Mathôt RA, OPTI‐CLOT Study Group and SYMPHONY Consortium, Cnossen MH (2022) Application of SHAP values for inferring the optimal functional form of covariates in pharmacokinetic modeling. Pharmacomet Syst Pharmacol 11(8):1100–1110. 10.1002/psp4.12828 PMC9381890 38100100

