# Supplementary material for: On inductive biases for the robust and interpretable prediction of drug concentrations using deep compartment models
Source: J Pharmacokinet Pharmacodyn. 2024 Mar 26;51(4):355–66. doi: 10.1007/s10928-024-09906-x (PMC11255087; doi:10.1007/s10928-024-09906-x)
Supplement: Supplementary file 1 — Supplementary file1 (DOCX 1822 kb) [file 10928_2024_9906_MOESM1_ESM.docx]

# Supplementary data 1 1A. Detailed description of model constraints

## Model constraints

We propose three simple approaches for constraining the solution space of DCMs (figure [2](#fig%25252525253Aconstraints)). First, boundary conditions were imposed on the PK parameters by using a transformed sigmoidal function following the output layer of the neural network (referenced as *boundary* constraint):

(S1)

Here, corresponds to the sigmoid function and is the constrained PK parameter vector. The boundaries can be set empirically based on prior knowledge. For example, bounds for the volume of distribution of drugs tightly bound to plasma proteins can be based on the expectation that the plasma volume of a typical male is roughly around 46 - 52 mL/kg [15]. We focus on setting a single boundary for all individuals, although it is possible to use a function to adapt **l** or **u** based on the covariates. Lower bounds of [0, 0.3, 0.05, 0] and upper bounds of [0.5, 7, 0.5, 2] for respectively CL (L/h), V_1_ (L), Q (L/h), and V_2_ (L) were used.

Next, global parameters **θ** for a subset of the PK parameters were estimated in parallel to *w* (referenced as *global parameters* constraint). We chose to estimate **θ** = {Q, V_2_} since these parameters affect the early distribution of FVIII, and drug concentration measurements at early time points are usually too sparse to identify covariate effects on these parameters. PK parameter vectors were reconstructed in the correct order using design matrices constructed using indicator functions **1**_A_. This function is specified in algorithm 1. An example of *A* = {1, 3} using one-based indexing results in:

(S2)

The PK parameter vector can then be reconstructed using the following equation (continuing the example of *A* = {1, 3}):

(S3)

Where -{*A*} corresponds to the indexes from .

Finally, we describe a neural network architecture where each covariate (or specific combinations thereof) are connected to independent sub-models *ψ*, whose predictions are combined using a product (referenced as the *multi-branch network*). This architecture is similar to a generalized additive model, using product accumulation rather than the sum of covariate effects. The use of a product ensures that **ζ**^(i)^ remains positive, regardless of the prediction from each sub-model as long as these are constrained to be positive. The use of a product also matches the standard implementation of covariates in population PK models (equation 3 in the main manuscript), and facilitates the interpretation of the clinical relevance of each covariate. For example, covariates resulting in a maximal net change 20% of the corresponding PK parameter are often deemed clinically insignificant in the pharmacometrics literature [16]. Again, indicator functions are used to determine the position of each prediction in the PK parameter vector:

(S4)

Here, **1**_0_ is an indicator function mapping zeros to ones. Softplus activation functions were used in the output layer of each sub-model. Single covariates can also be linked to multiple PK parameters. Likewise, multiple covariates can be passed to a sub-model when an interaction between covariates is expected. An added benefit of this approach is that the output of each sub-model can be visualized, allowing for the interpretation of the learned covariate effects. A schematic overview of the multi-branch network is provided in supplementary figure [5](#suppfig%25252525253Amulti-branch-schemat).

# 1B. Detailed investigation of effects of constraints on model training.

In this section, we further investigate the effect of each constraint on model training. First, we found that there was roughly 4.5x greater variability in median RMSE over replicates over data folds compared to within fold replicates. This suggest that weight initializations have a relatively small effect on final model accuracy. Looking at the number of divergent models when fitting the naive model, the frequency of divergent models seemed to be related to specific data folds:


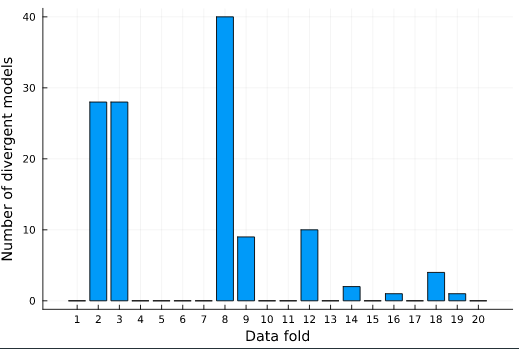


In 21/36 of examples where divergent models occurred, 4 or 5 of the within fold replicates resulted in divergent models. Most divergent models were trained on data from fold 2, 3, and 8. We therefore look specifically into the distribution of the covariates as well as the initial PK parameter estimates for the models trained on these folds.

We did not observe very large differences between covariate distributions, with possibly a slightly higher fraction of younger patients in data folds 2, 3 and 8. This might cause the model to lean slightly more towards lower volume of distributions (as children have lower volume of distribution), resulting in sharper peaks. There do not seem to be very distinct differences between the drug levels between ‘bad’ and ‘good’ data folds:


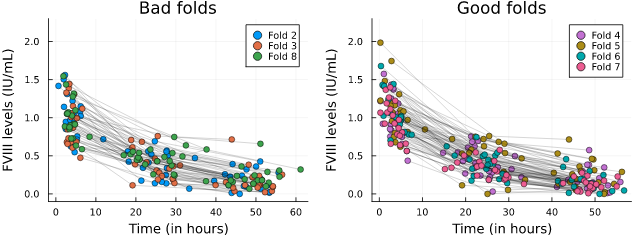


Random initialization of the naive neural network weights results in initial estimates of the PK parameters around 0.7 (L or L/h):


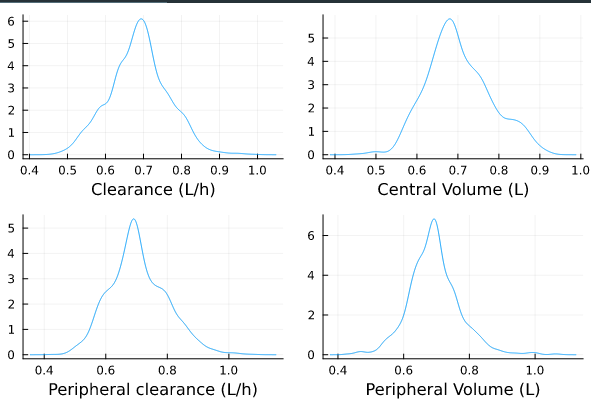


This image shows 100 random initialization of the network weights and the distribution of resulting initial PK parameter estimates for subjects in training fold 2 with n = 120. The distributions are data agnostic, they initialize around 0.7 no matter the input data. Due to the relatively low initial estimate for V_1_ and high estimate for CL, concentration time curves start out with high peak concentrations and short half-life.

Next we look at how the PK parameters change during optimization when training on n = 120 and n = 20 on one of the ‘problematic’ folds (and compare to n=20 on a ‘good’ fold)
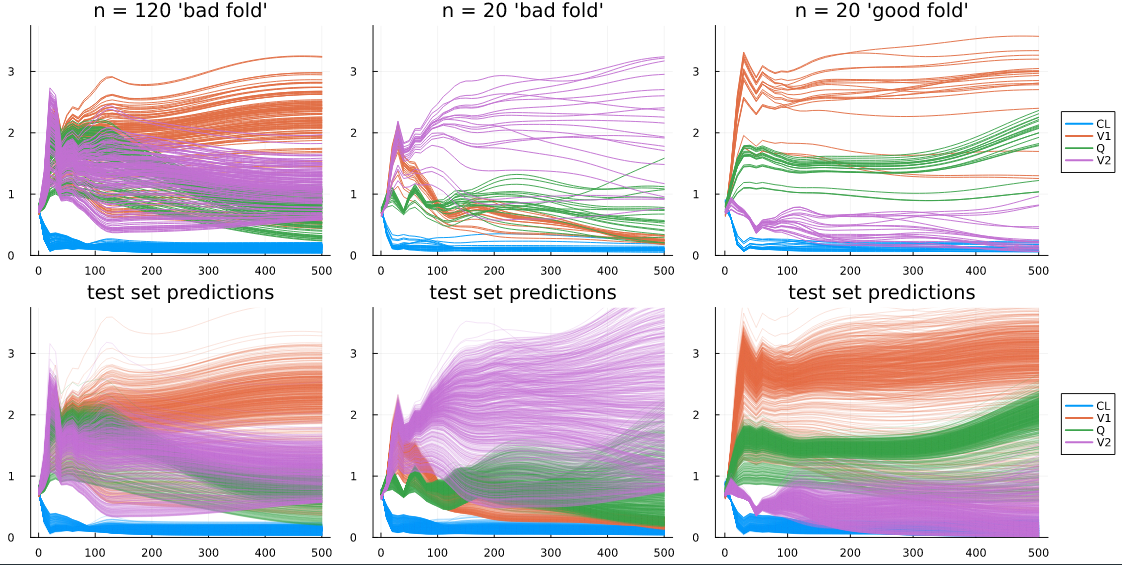
:

We see that for one of the problematic folds (fold 2) the estimate of volume of distribution (V_1_) drops down towards zero after an initial increase for all subjects at n = 20, while V_2_ increases rapidly. This results in high peak concentration predictions due to a rapid distribution from a small central volume into a larger peripheral volume. This is not seen when training on larger patient data sets, or on ‘good’ data folds.

Removing the younger patients from the data fold is not sufficient to improve optimization:


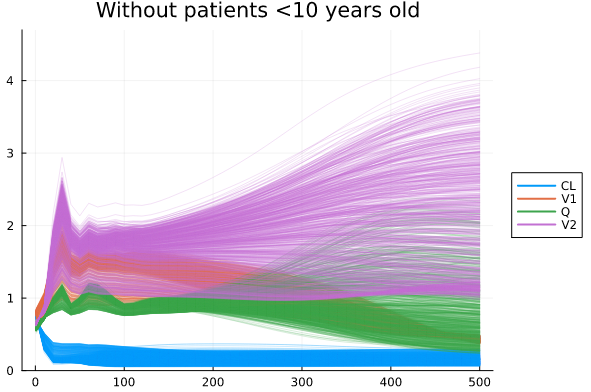


We then look at the effect of adding constraints to the model:


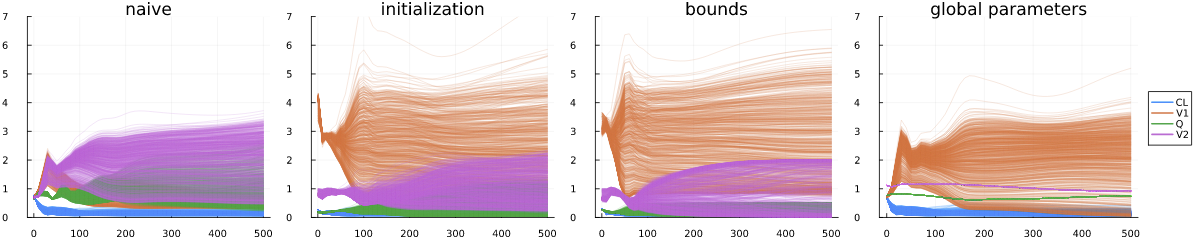
Above we show the PK parameter predictions after training on n = 20 subjects for a ‘bad’ fold, and show predictions in the test set. Similar to initialization, the addition of bounds to the value of the PK parameters changes the initial estimates of the PK parameters. The effect of initialization and providing bounds seem to be somewhat similar, with a notable exception that placing bounds causes the estimates of V_2_ to be mainly located at the extremes of the bound (i.e. 0 and 2 L). Additionally, the variability in V_1_ is larger compared to the other models. It is possible that placing bounds seems to have a similar effect as initialization.

In the global parameter model, PK estimates during the first 50 epochs of training look somewhat similar to those obtained using the naive model:


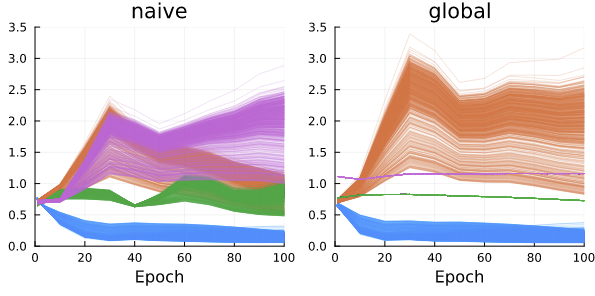


An important distinction is that in the naive model, the estimate for V_2_ rises concurrently with the value of V_1_, whereas in the global parameter model, V_2_ remains somewhat stable during optimization. It seems that setting global variables regularizes the optimization procedure in such a way that gradients of these parameters are potentially smaller compared to the other parameters.

We can show that specifically using a global variable for V_2_ has a similar effect as using global parameters for both parameters. Using a global parameter for Q still results in unrealistic solutions.


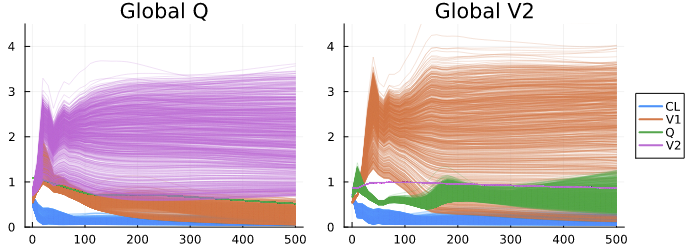


If we set the initial estimates of V_2_ to be very high (3 L) or low (0.1 L) the model still results in reasonable models when using global parameters for V_2_:


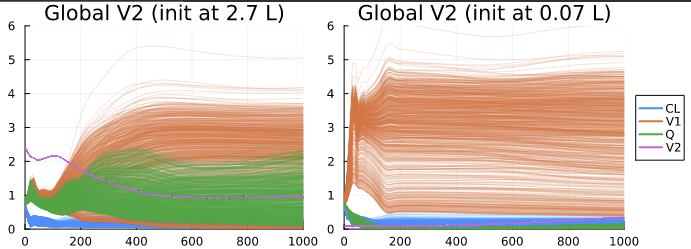


Optimization can also be improved by only setting bounds on the value of V_2_:


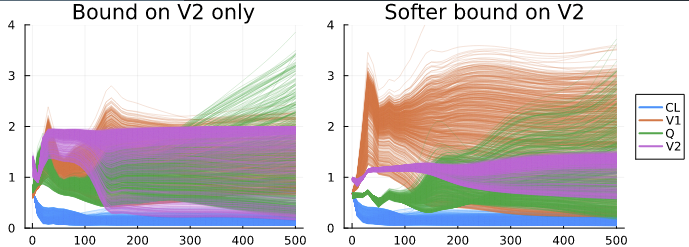


We find here that placing softer bounds (for example by using a softsign instead of a sigmoid) might be a reasonable approach, since we observe a propensity of V_2_ estimates ‘getting stuck’ at the extremes of the sigmoid during optimization.

We ran the synthetic experiment again for these two V_2_ specific constraints on the data sets with *n* = 20 subjects, using 8 neurons in the hidden layer of the neural network:

| Model | Median RMSE ± one SD (%-age divergent) | |
| --- | --- | --- |
|  | Weight + Height + Age | FFM + Age |
| Naive (from original experiment) | 14.7 ± 0.42 (18%) | 14.1 ± 0.48 (12%) |
| Softsign bound on V_2_ | 17.4 ± 3.5 (5%) | 17.5 ± 21.8 (5%) |
| Global parameter for V_2_ | 16.8 ± 2.1 (2%) | 16.4 ± 41.1 (1%) |

**Conclusion**

The results hint at the importance of the data used for training (especially when the number of samples is sparse), since it can bias the optimization procedure to converge to unrealistic solutions. Unfortunately, we could not find specific data that causes the behaviour.

Although initialization and the use of bounds seem to improve optimization, there is still a large variability in the PK parameters after convergence. Global parameters on the other hand seem to result in distinct solutions, which we found (in this case) to be more similar to models trained on larger data sets. We can further identify the behaviour to be specifically related to the estimate of V_2_. Setting this PK parameter to be global, or setting bounds on it specifically can improve solutions. It could be the case that the models have identifiability issues between V_1_ and V_2_ during optimization, such that additional constraints can potentially be useful to improve the model.
