# Supplementary material for: On inductive biases for the robust and interpretable prediction of drug concentrations using deep compartment models
Source: J Pharmacokinet Pharmacodyn. 2024 Mar 26;51(4):355–66. doi: 10.1007/s10928-024-09906-x (PMC11255087; doi:10.1007/s10928-024-09906-x)
Supplement: Supplementary file 2 — Supplementary file2 (DOCX 6 kb) [file 10928_2024_9906_MOESM2_ESM.docx]

# Supplementary data 2: Visualization of covariate effects

Visualization of the learned effects for each of the covariates were obtained from the multi-branch networks by taking each sub-model and entering dummy input within the domain of the training data. In order to compare learned effects across model replicates, the output of each sub-model $\psi_{s}$ was normalized with respect to its prediction at the location of the median covariate value:

$\psi_{s}\left( x_{s} \right)=\frac{\psi_{s}\left( x_{s} \right)}{\psi_{s}\left( Med\left[ x_{s} \right] \right)}$ (S5)

The prediction of $\vec{\zeta}^{\left( i \right)}$ now decomposes into:

$\zeta_{m}\left( \vec{x} \right)=\theta_{TV}\cdot\prod_{s}^{S_{m}} \psi_{s}\left( x_{s} \right)$ (S6)

Where $\theta_{TV}=\prod_{s}^{S_{m}} \psi_{s}\left( Med\left[ x_{s} \right] \right)$ is the typical value for PK parameter $m$ over all individuals. Note the similarity of this equation to equation 3 in the main manuscript. This way the prediction from each neural network is anchored to 1 at the median value of each of the covariates, similar to how covariates are implemented in NONMEM. Since covariate effects are combined using a product, the full model is unconstrained with respect to the scale of the predictions from each sub-model and variance of the unnormalized learned effects is high. For example, if the prediction of $\psi_{1}$ is very low after random initialization of the network, the model can still produce the same PK parameter predictions to other replicates by increasing the scale of predictions from $\psi_{2}$. The normalization corrects for these differences between replicates.

Now that we have $\psi_{s}\left( x_{s} \right)$, we can query this model to obtain predictions at any value of the covariate. Visualization of these predictions results in the figures as reported in the manuscript.
