# Supplementary material for: On inductive biases for the robust and interpretable prediction of drug concentrations using deep compartment models
Source: J Pharmacokinet Pharmacodyn. 2024 Mar 26;51(4):355–66. doi: 10.1007/s10928-024-09906-x (PMC11255087; doi:10.1007/s10928-024-09906-x)
Supplement: Supplementary file 3 — Supplementary file3 (DOCX 380 kb) [file 10928_2024_9906_MOESM3_ESM.docx]

# Supplementary tables and figures

**
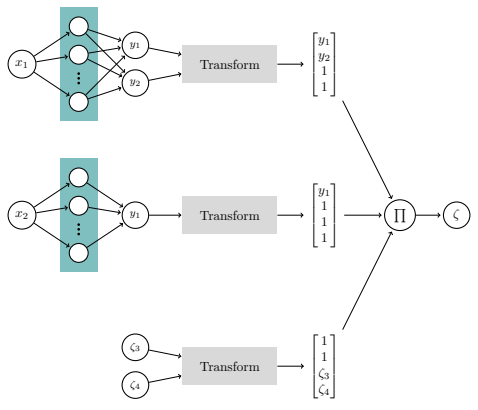
**

**Figure s1. Schematic representation of the multi-branch network.**

**Table s1. Results for the models trained using a hidden layer size of 32.**

|  | **Mean RMSE ± one SD (percentage divergent)** | | |
| --- | --- | --- | --- |
| **Weight, height, age** | $n=20$ | $n=60$ | $n=120$ |
| None | 15.7 ± 32 (17) | 12.9 ± 4.5 (4) | 12.1 ± 0.39 (0) |
| Initialization | 16.7 ± 19 (8) | 13.0 ± 4.6 (4) | 12.2 ± 0.69 (0) |
| Boundary | 15.8 ± 3.0 (2) | 12.7 ± 0.81 (0) | 12.1 ± 0.78 (0) |
| Global parameters | 14.1 ± 5.7 (1) | 12.8 ± 0.43 (0) | 12.1 ± 0.27 (0) |
| **FFM, age** |  |  |  |
| None | 14.3 ± 49 (18) | 12.6 ± 1.6 (3) | 12.1 ± 0.32 (0) |
| Initialization | 15.1 ± 26 (9) | 12.6 ± 2.8 (5) | 11.9 ± 0.37 (0) |
| Boundary | 14.2 ± 2.1 (2) | 12.5 ± 0.34 (0) | 11.9 ± 0.3 (0) |
| Global parameters | 13.3 ± 0.87 (0) | 12.6 ± 0.33 (0) | 12.0 ± 0.28 (0) |

RMSE = root mean squared error, SD = standard deviation

**Table s2. Results for the models trained using a hidden layer size of 128.**

|  | **Mean RMSE ± one SD (pergentage divergent)** | | |
| --- | --- | --- | --- |
| **Weight, height, age** | $n=20$ | $n=60$ | $n=120$ |
| None | 16.8 ± 36 (21) | 13.0 ± 8.1 (5) | 12.1 ± 0.58 (0) |
| Initialization | 16.5 ± 31 (6) | 13.2 ± 9.7 (5) | 12.4 ± 0.87 (0) |
| Boundary | 15.8 ± 2.2 (2) | 12.8 ± 0.63 (0) | 12.2 ± 0.93 (0) |
| Global parameters | 14.1 ± 1.3 (0) | 13.0 ± 0.57 (0) | 12.2 ± 0.39 (0) |
| **FFM, age** |  |  |  |
| None | 15.5 ± 41 (24) | 12.6 ± 4.6 (5) | 12.1 ± 0.43 (0) |
| Initialization | 15.4 ± 16 (13) | 12.6 ± 9.1 (5) | 12.0 ± 0.4 (0) |
| Boundary | 14.7 ± 1.4 (0) | 12.6 ± 0.42 (0) | 12.0 ± 0.37 (0) |
| Global parameters | 13.5 ± 0.85 (0) | 12.5 ± 0.43 (0) | 12.0 ± 0.27 (0) |

RMSE = root mean squared error, SD = standard deviation


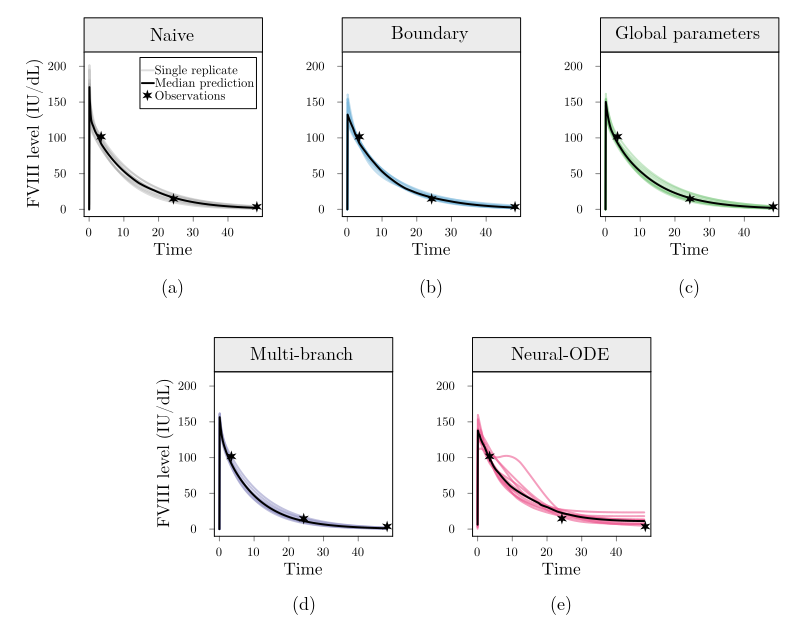

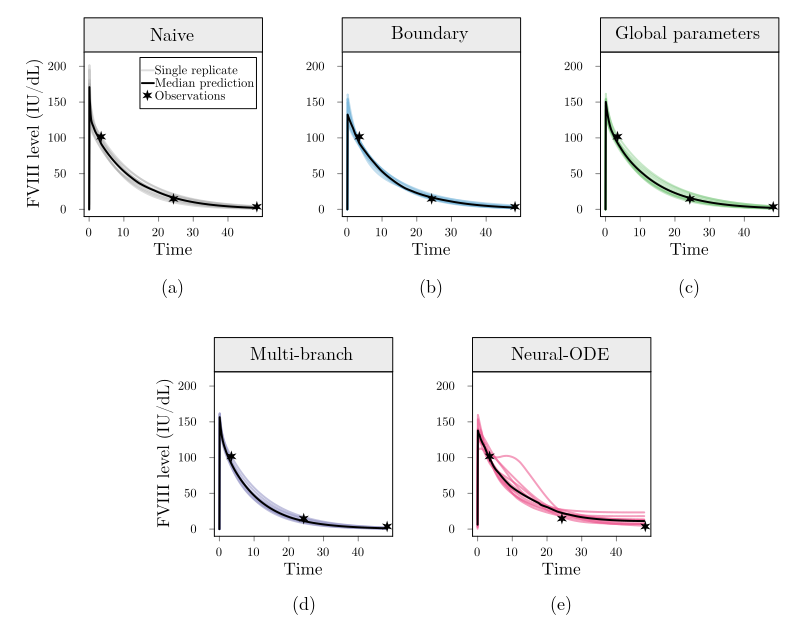


**Figure s2. Comparison of the predicted concentration-time curves in the real-world data experiment.**Results are shown for the naive (a), boundary constraint (b), global parameter (c), multi-branch network (d), and Neural-ODE (e) models. The median prediction (black line) over the 10 data set folds (lightly coloured lines) along with the observations (stars) are shown for the same patient.


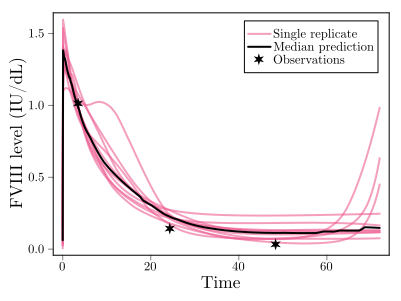


**Figure s3. Extrapolation of predictions in the Neural-ODE quickly degenerate.**Showing the prediction for the same patient as in Figure s2, but with an expanded time window. Predictions by the Neural-ODE can behave unexpectedly when data is insufficient to fully describe drug kinetics. The median prediction (black line) over the 10 data set folds (lightly coloured lines) along with the observations (stars) are shown.


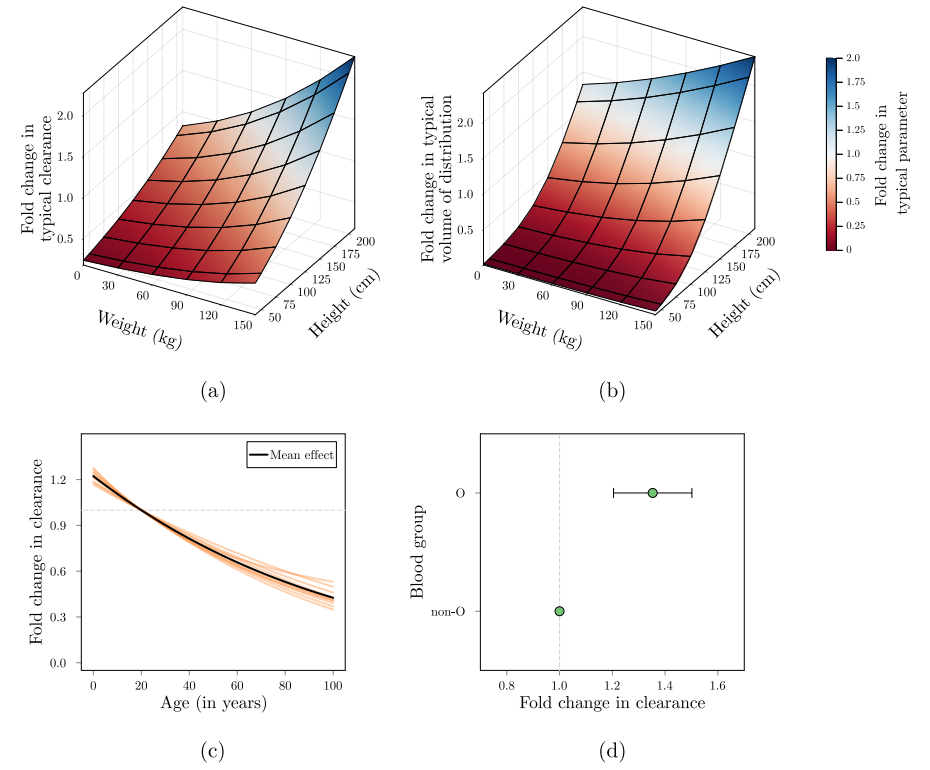


**Figure s4. Learned effects of the multi-branch network in the real-world data experiment.**

In the top panel, the combined effect of weight and height on clearance and volume of distribution are shown. Horizontal and vertical lines depict the marginal effect of respectively weight or height at a fixed value of the other covariate. In a, we can see that weight and height are similarly important for predicting changes in clearance. However, in b we see that the importance of height is stronger than that of weight. In the bottom panel, the effect of age on clearance (c) and blood group on clearance (d) are shown.
